# Prmt5 differentiates developmental *vs* regenerative myogenesis

**DOI:** 10.18632/oncotarget.6207

**Published:** 2015-10-21

**Authors:** Ting Zhang, Thomas Braun, Yonggang Zhou

**Affiliations:** Max-Planck-Institute for Heart and Lung Research, Department of Cardiac Development and Remodeling, Bad Nauheim, Germany

**Keywords:** Chromosome Section, myogenesis, MuSC, muscle progenitor cell, Prmt5, epigenetics

Skeletal muscle, the most abundant tissue in mammal body, is indispensable for locomotion but also plays a major role in the regulation of energy metabolism. Muscle fibers are mainly generated during embryonic and fetal development, yet physiological postnatal muscle enlargement depends mostly on hypertrophic growth. Muscle regeneration after injury critically depends on activation and proliferation of muscle stem cells (MuSC), also called satellite cells. Developmental myogenesis relies on proliferation and differentiation of mesodermal progenitor cells that become specified by expression of myogenic regulators of the MyoD/Myf5 transcription factor family. During development Pax7-positive muscle progenitor cells are set aside by unknown mechanism and later form the MuSC pool. It is generally assumed that regenerative myogenesis recapitulates most aspects of developmental myogenesis although it seems likely that important differences exist [1]. In fact, muscle progenitor cells and MuSC express similar sets of regulatory factors and activate similar signaling pathways during proliferation and differentiation to muscle fibers. Prominent examples include the muscle regulatory factors (MRFs) such as MyoD, Myf5, MRF4 or MyoG, all of which follow similar sequential expression patterns [2]. On the other hand, muscle progenitor cells and MuSC differ dramatically in terms of cell proliferation and chromatin organization. Embryonic muscle progenitor cells are highly proliferative responding to the need to rapidly build the skeletal muscle apparatus. In contrast, MuSC are mostly quiescent under physiological conditions and show only a low turnover rate. Muscle progenitor cells and MuSC are also characterized by a different organization of the heterochromatin indicating that epigenetic regulations have a differential impact on either cell type [3]. In line with these arguments, we recently identified an epigenetic modifying enzyme called protein arginine methyltransferase 5 (Prmt5), which is required for MuSC proliferation but dispensable for expansion of muscle progenitor cells [4].

Prmt5 was identified in a functional shRNA screen of molecules that were enriched at the protein level in MuSC. Characterization of Prmt5 by loss and gain of function assays *in vitro* followed by genetic inactivation *in vivo* revealed a crucial function for MuSC proliferation and differentiation. Inactivation of Prmt5 in MuSC resulted in abrogation of muscle formation after acute and chronic muscle injury demonstrating a critical role of Prmt5 in control of adult muscle regeneration. Molecular studies indicated that Prmt5 epigenetically silences the cell cycle inhibitor p21 by methylation of histone H3R8. Interestingly, deletion of p21 partially restored proliferation of Prmt5 deficient MuSC suggesting that repression of p21 by Prmt5 is critical to enable proliferation of MuSC. Surprisingly, inactivation of Prmt5 in Pax7-positive muscle progenitor cells during embryonic development showed no obvious defects indicating fundamental differences between embryonic and adult myogenesis, which are -at least in part-mediated by Prmt5 [4].

Muscle progenitor cells and MuSC are located in different microenvironments or niches and respond to distinct external myogenic stimuli. Such differences might be transformed to distinct nuclear responses leading to activation of different transcriptional programs and/or different chromatin organization. Prmt5 seems to be a positive component of the nuclear epigenetic regulatory network that silences cell cycle regulators thereby keeping adult MuSC in a primed state, ready for external stimulation and proliferation. The prevalent location of Prmt5 in the cytoplasm during embryonic development suggests that Prmt5 plays only a minor role in the epigenetic regulation of gene expression. Instead Prmt5 might regulate cytoplasmic signaling cascades although this function does not seem to be dominant during developmental myogenesis [5]. One potential signaling cascade that might be affected by the action of Prmt5 in the cytoplasm is JAK-STAT pathway, since Prmt5 was initially identified as an interaction partner of JAK and negatively regulates this signaling cascade in cancer [6]. The differential requirement of Prmt5 for proliferation of embryonic and adult muscle cells makes a lot of sense since regulation of quiescence and proliferation but not lineage commitment and differentiation differs dramatically between the embryonic and adult states. One obvious question for the future is the analysis of the Prmt5-independent control of embryonic muscle progenitor cell proliferation. Since rhabdomyosarcomas are typical childhood tumors [7] the tantalized possibility emerges that the differential regulation of muscle cell proliferation and the different function of Prmt5 at different developmental stages have a major, so far underestimated role in the development of this disease.
